# Formula for Pepsin Wine

**Published:** 1860-05

**Authors:** 


*Formula for Pepsine Wine.*—Take of starchy pepsine prepared according to MM. Corvisart and Boudault's formula, one drachm and a half; distilled water, six drachms; white wine (of Lunel) fifteen drachms; white sugar, one ounce; spirit of wine (33°) three drachms; mix until the sugar is quite dissolved and filter. One table-spoonful of this contains fifteen grains of pepsine, and may be given after every meal.

